# Alphavirus replicon particles containing the gene for HER2/*neu *inhibit breast cancer growth and tumorigenesis

**DOI:** 10.1186/bcr962

**Published:** 2004-11-29

**Authors:** Xiaoyan Wang, Jian-Ping Wang, Maureen F Maughan, Lawrence B Lachman

**Affiliations:** 1 Department of Bioimmunotherapy, The University of Texas MD Anderson Cancer Center, Houston, Texas, USA; 2 The Graduate School of Biomedical Sciences, The University of Texas Health Sciences Center, Houston, Texas, USA; 3 AlphaVax, Inc., Research Triangle Park, North Carolina, USA

**Keywords:** adjuvant treatment, breast cancer, gene vaccines, immunotherapy, virus-like replicon particles

## Abstract

**Introduction:**

Overexpression of the HER2/*neu *gene in breast cancer is associated with an increased incidence of metastatic disease and with a poor prognosis. Although passive immunotherapy with the humanized monoclonal antibody trastuzumab (Herceptin) has shown some effect, a vaccine capable of inducing T-cell and humoral immunity could be more effective.

**Methods:**

Virus-like replicon particles (VRP) of Venezuelan equine encephalitis virus containing the gene for HER2/*neu *(VRP-*neu*) were tested by an active immunotherapeutic approach in tumor prevention models and in a metastasis prevention model.

**Results:**

VRP-*neu *prevented or significantly inhibited the growth of HER2/*neu*-expressing murine breast cancer cells injected either into mammary tissue or intravenously. Vaccination with VRP-*neu *completely prevented tumor formation in and death of MMTV-c-*neu *transgenic mice, and resulted in high levels of *neu*-specific CD8^+ ^T lymphocytes and serum IgG.

**Conclusion:**

On the basis of these findings, clinical testing of this vaccine in patients with HER2/*neu*^+ ^breast cancer is warranted.

## Introduction

The management of breast cancer currently relies on surgery, chemotherapy and radiotherapy. Despite recent advances in clinical management of breast cancer once metastasis has occurred, the probability of a complete cure is greatly reduced. Of the women who have no detectable lymph node metastases at the time of diagnosis, up to one-third later develop metastases [1]. In patients with metastatic disease that does not respond to radiotherapy or chemotherapy, immunotherapy may offer an additional form of cancer control [2-4]. Clinical trials of trastuzumab, a monoclonal antibody specific for HER2/*neu*, have demonstrated the utility of an immunologic approach for breast cancers that overexpress this gene [5-7]. A drawback to 'passive' immunotherapy using monoclonal antibodies is that the effect is short-lived. An alternative approach is active vaccination that could induce *neu*-specific cytotoxic T cells with the ability to control the growth of the primary tumor and metastases. However, unlike passive immunotherapy whose effectiveness quickly wanes, effector and memory T cells induced by vaccination may remain present and be able to respond to any metastatic cells expressing HER2/*neu *that arise after treatment.

HER2/*neu *is an excellent target for gene vaccines, and several preclinical studies have shown the effectiveness of plasmid vaccines encoding *neu *in murine models [8-16]. Using a plasmid markedly different from those previously described [8-16], we created an effective gene vaccine against HER2/*neu *[17]. The previously described ELVIS plasmid vaccine construct for HER2/*neu *contained the cDNA of a replicon RNA from the Alphavirus Sindbis [18,19]. The replicon RNA contained the replication/transcription genes of the parent virus, but the structural protein genes were replaced by the gene for rat *neu*. From this plasmid construct, the replicon RNA is synthesized in the nucleus of the host cell and is transported to the cytoplasm for replication and transcription. The *neu *gene product is produced at high levels in the cytosol. Since the structural protein genes from the parent virus are not encoded by the replicon, progeny infectious Sindbis virions are not generated [20-22].

Alternatives to Alphavirus replicon plasmid vaccines [17,19,23-25] are Alphavirus-based virus-like replicon particles (VRP). As already mentioned, the replicon RNA does not contain the structural genes from the parent virus. It is therefore a single-cycle, propagation-defective RNA and replicates only within the cell into which it is introduced. To generate VRP from an attenuated strain of the Alphavirus Venezuelan equine encephalitis virus (VEE), the replicon RNA is packaged into particles by co-transfecting the replicon RNA and two separate helper RNAs, which together encode the full complement of VEE structural proteins [26]. Although the VRP can infect target cells in culture or *in vivo*, and can express the foreign gene to a very high level, the VRP are defective since they lack critical portions of the VEE genome – they lack the VEE structural protein genes necessary to produce infectious virus particles capable of spreading to other cells [21,27].

Several reports have demonstrated that VRP are extremely effective vaccine vectors [28-39]. The VEE VRP vaccine vectors are particularly attractive because the VEE envelope glycoproteins target the VRP to cells of lymphoid tissue [40], because they can be administered multiple times [39], because they induce both cellular and humoral immune responses, and because pre-existing immunity to VEE in humans should not be problematic since the incidence of VEE infection is low.

In the current study, we sought to determine whether vaccination with VEE-derived VRP containing the gene for HER2/*neu *would inhibit tumor growth in prevention models in which HER2/*neu*-expressing tumor cells had been injected either into a mammary fat pad or intravenously. We also sought to determine whether vaccination could prevent spontaneous tumorigenesis in HER2/*neu *transgenic mice. VRP-*neu *vaccination induced antigen-specific CD8^+ ^T-cell and IgG responses that corresponded with the lack of tumor growth in both tumor models. In light of the clinical benefit of trastuzumab, a safe and effective vaccine that can induce cellular and humoral immunity, VRP-*neu *warrants clinical evaluation.

## Materials and methods

### Tumor cell line and reagents

The A2L2 cell line that expresses high levels of rat HER2/*neu *has been previously described in detail [17]. The A2L2 cell line has consistently expressed high levels of HER2/*neu *for more than 5 years and consistently induces tumors in Balb/c mice when injected into a mammary fat pad or intravenously. The A2L2 cell line was maintained in Eagle's MEM containing 5% FCS, sodium pyruvate, nonessential amino acids, L-glutamine, and vitamins (GIBCO, Carlsbad, CA, USA). The monolayer cultures were subdivided at approximately 75% confluence by treatment for 1–3 min with 0.25% trypsin and 0.02% EDTA at 37°C.

### Virus-like replicon particles

The pSV2-*neu *plasmid containing the gene for rat HER2/*neu *was provided by Dr Mien-Chie Hung (The University of Texas MD Anderson Cancer Center, Houston, TX, USA). VEE VRP were constructed at AlphaVax, Inc. (Research Triangle Park, NC, USA) according to the published procedure [26]. Control VRP containing the gene for A/PR/8/34 influenza hemagglutinin (HA) were also prepared following the same procedure. VRP preparations were screened in a sensitive *in vitro *Vero cell cytopathic effect assay for the detection of replication competent virus. Prior to their use in these studies, the preparations were shown to be devoid of detectable replication competent virus.

### Flow cytometry

A2L2 cells were incubated for 1 hour at 37°C with either immune serum or control serum diluted in PBS. FITC-labeled goat anti-mouse IgG diluted 1:1000 in PBS was added to the cells and incubation was continued for 1 hour at 37°C. The cells were washed three times in PBS and were analyzed by flow cytometry using an EPICS Profile Analyzer (Beckman Coulter, Inc., Fullerton, CA, USA).

### Mice

Female Balb/c mice, aged 6–8 weeks, were obtained from the Frederick Cancer Research Facility (Frederick, MD, USA). Mice were allowed to acclimate for at least 1 week before use. Six-week-old MMTV-c-*neu *transgenic mice were obtained from Charles River Laboratories Inc. (Wilmington, MA, USA). This strain of mice is called the 'Oncomouse' by Charles River Laboratories due to the spontaneous generation of breast cancer. These mice are of the FVB/N strain, and the transgene is the 'activated' rat c-*neu *oncogene preceded by the MMTV promoter [41].

### Vaccination with VRP

Mice were vaccinated subcutaneously in the hind foot pad with VRP suspended in 50 μl normal saline. Repeat vaccinations, when administered, were performed using alternate hind feet. Tail vein blood was removed and tumor challenge performed 2 weeks after the final vaccination. Serum was separated from the blood by centrifugation after overnight storage at 4°C.

### Mammary fat pad tumor prevention model

A2L2 cells were harvested from subconfluent cultures with trypsin and EDTA as described earlier. The cells were washed once in serum-containing culture medium and were washed once in PBS. Mice were anesthetized by inhalation of isoflurane using a special apparatus developed by the veterinarians at MD Anderson Cancer Center. The fur was shaved over the lateral thorax, and a 5-mm-long incision was made to reveal mammary fat pad 2 as previously described [42]. A 0.1-ml sample containing 2.5 × 10^4 ^A2L2 cells in normal saline was injected into the fat pad. The incision was closed with a wound clip. Wound clips that had not already fallen off were removed after 7 days. The mice were then observed daily and their tumors measured in perpendicular directions with a pair of calipers. Mice with tumors 10 mm in the greatest dimension were killed according to our approved Institutional Animal Care and Use protocol. At the termination of the experiment, all mice were killed by CO_2 _inhalation and all tumors were excised and weighed.

### Experimental metastasis prevention model

A 0.1-ml sample containing 2.5 × 10^4 ^A2L2 cells in normal saline was injected into the tail vein of each immunized mouse. The mice were killed 30 days later, and the surface lung metastases in each animal were counted.

### Tetramer analysis of immune spleen cells

A K(d) tetramer containing the peptide sequence PYVSRLLGI [43] was prepared by the National Institutes of Health Tetramer Facility at Emory University (Atlanta, GA, USA). The incorporated peptide was synthesized at the peptide synthesis facility of MD Anderson Cancer Center. We received a K(d) tetramer specific for A/PR/8/34 influenza HA containing the peptide IYSTVASSL from Linda Sherman at the Scripps Research Institute (La Jolla, CA, USA) [44]. A single-cell suspension of the spleen from a naïve mouse was prepared in R10S medium (RPMI 1640, 10% HI-FCS [Summit Biotechnology, Fort Collins, CO, USA], 1% nonessential amino acids, 100 mM MEM sodium pyruvate, 2 mM L-glutamine, 100 U/ml penicillin, 1 ml streptomycin, and 50 mM 2-mercaptoethanol) by gently swirling the spleen between frosted glass slides.

Debris was removed from the cell suspension by filtration through nylon mesh into a 10-ml centrifuge tube. The cell suspension was centrifuged for 10 min at 700 × *g *and the pellet resuspended in 5 ml ACK solution (0.15 M NH_4_Cl, 1.0 mM KHCO_3_, 0.01 mM NaEDTA, pH adjusted with 1 N HCl to 7.2–7.4) before being gently rocked for 5 min to lyse red blood cells. An additional 5 ml R10S medium was added to the cell suspension, and the cells were washed once with R10S medium. The naïve spleen cells were cultured with either PYVSRLLGI or IYSTVASSL (70 ng/ml) on a rocker for 2 hours at 37°C and were γ-irradiated with 20 Gray.

The mice were vaccinated three times with a 2-week interval with 10^6 ^infectious units (IU) VRP-*neu *or 10^6 ^IU VRP-HA, and the immune spleens were harvested 3 weeks after the final vaccination. The immune spleens were prepared to produce a single cell suspension in R10S medium and were co-cultured with the peptide-pulsed stimulator cells at a ratio of one stimulator to eight responders. The cell mixture was cultured for 7 days at 37°C, washed, and was resuspended in FACS buffer (PBS with 1% BSA) at a concentration of 5 × 10^7 ^cells/ml. Twenty microliters of the cell mixtures were added to 20 μl PE-conjugated Her2/*neu-*specific or HA-specific tetramers to make the final dilutions of the tetramers 1:50, 1:100, and 1:200. One microliter of PerCP-conjugated anti-mouseCD3e (PharMingen, San Diego, CA, USA) and FITC-conjugated anti-mouse CD8a (Caltag, Burlingame, CA, USA) was added to the mixture and incubated for 1 hour at 4°C in the dark. The cells were then suspended in 150 μl FACS buffer and transferred to a polystyrene round-bottomed tube. The cells were washed twice with FACS buffer and suspended in 200 μl of 1% paraformaldehyde in PBS.

List mode data were acquired with a FACScan (BD Biosciences, San Jose, CA, USA). Dead cells and monocytes were excluded from the analysis by forward scatter and side scatter gating. A total of 10,000–30,000 events were typically acquired, and compensation was optimized using unstained cells, cells stained with only PerCP-conjugated anti-mouse CD3e, cells stained with only FITC-conjugated anti-mouseCD8a and cells stained with only PE-conjugated anti-mouse CD4. The CD3^+ ^cells were gated from the total population of live cells, and the CD8^+ ^cells were gated from the CD3^+ ^cells. From the CD3^+ ^cells and the CD8 double-positive cells, the percentages of PE-tetramer-positive cells were calculated. Isotype controls for the anti-CD3e and anti-CD8a were subtracted from the acquired data. List mode files were analyzed using CELLQUEST software (BD Biosciences, San Jose, CA, USA).

### Intracellular interferon-γ analysis of immune spleen cells

The procedure for this technique was obtained from PharMingen, whose reagents were used whenever possible. Spleens from VRP-*neu*-immunized and VRP-HA-immunized mice were prepared as already described for tetramer analysis. The spleen cells were stimulated with PYVSRLLGI at a concentration of 70 ng/ml at 37°C in R10S medium for 5 days as described. On the sixth day, the cells were suspended at 2 × 10^6^/ml in R10S medium containing 10 ng/ml phorbol-12-myristate acetate (Sigma-Aldrich, St Louis, MO, USA) and 250 ng/ml ionomycin (Sigma-Aldrich). Brefeldin (1 μl/ml; Sigma-Aldrich) was added at the same time to block cytokine secretion. The cells were washed after 5 hours and resuspended at 10^7 ^cells/ml in staining buffer (Dulbecco's PBS without Mg^2+^or Ca^2+^, 1% heat-inactivated FCS, w/v 0.09% sodium azide; pH adjusted to 7.4–7.6). The cells were then incubated for 15 min with purified 2.4 G2 antibody 10 μg/ml (PharMingen) to block nonspecific staining by fluorochrome-conjugated antibodies via Fc receptors. The cells were washed twice with staining buffer, and 10^6 ^cells were stained with 1 μg FITC-labeled anti-mouse CD8a and PerCP-labeled anti-mouse CD3e (PharMingen) in 50 μl staining buffer at 4°C for 30 min. The cells were again washed twice with staining buffer, followed by fixation and permeabilization with Perm/Wash (Cytofix/Cytoperm Kits; PharMingen) for 20 min at 4°C. The cells were washed twice and resuspended in 50 μl of the same solution. PE-conjugated anti-interferon (IFN)-γ monoclonal antibody (0.5 μg/10^6 ^cells; PharMingen) was added and the suspension was incubated for 30 min at 4°C. The cells were then washed twice with Perm/Wash solution and resuspended in staining buffer.

The FACScan was used to analyze the percentage of intracellular IFN-γ-containing cells among the CD3^+ ^and CD8^+ ^cells. Isotype controls for anti-CD3a, anti-CD8e, and anti-IFN-γ (PharMingen) were subtracted from the acquired data.

### ELISPOT analysis of IFN-γ production by immune spleen cells

The procedure for the ELISPOT technique was obtained from PharMingen, whose reagents were used whenever possible. The wells of an ELISPOT plate (CTL Immunospot plate; Cellular Technology Ltd, Cleveland, OH, USA) were coated overnight at 4°C with 100 μl anti-mouse IFN-γ (2 μg/ml; PharMingen) diluted at 1:200 in coating buffer (PBS, pH 7.2). The coated wells were washed with blocking solution (R10S medium), fresh blocking solution was added to each well, and then the plate was incubated for 2 hours at room temperature. The blocking solution was then discarded and 100 μl A2L2 cells (2 × 10^5^-1 × 10^6^) was added to each well of the ELISPOT plate.

Immune spleens were dissected from vaccinated mice and were prepared as already described for tetramer analysis. Increasing numbers of spleen cells (5 × 10^6 ^- 2 × 10^7^) in 100 μl R10S medium were added to the wells, and the plate was incubated for 24 hours at 37°C in a 5% CO_2 _atmosphere at 99% humidity. Wells containing only spleen cells served as negative controls, and spleen cells from VRP-*neu*-vaccinated mice cultured overnight with 5 μg/ml concanavalin A (Sigma-Aldrich) served as a positive control. The cell suspension was aspirated, and the wells were washed twice with deionized water and were then soaked with deionized water for 5 min. The wells were washed three times with wash buffer I (PBS containing 0.05% Tween-20).

The detection antibody, biotinylated anti-mouse IFN-γ (PharMingen), was diluted to 2 μg/ml in dilution buffer (PBS containing 10% FBS), and 100 μl was added to each well. The plate was incubated at room temperature for 2 hours, and the wells were washed three times with buffer I. Avidin–horseradish peroxidase reagent (PharMingen) was diluted to 1:100 in dilution buffer, and 100 μl was added to each well, which was then incubated at room temperature for 1 hour. The wells were washed four times with wash buffer I and twice with wash buffer II (PBS). A stock solution containing 100 mg 3-amino-9-ethyl-carbazole (Sigma) dissolved in 10 ml *N*, *N*-dimethylformamide (Sigma-Aldrich) was prepared. The final substrate solution was made by adding 333 μl 3-amino-9-ethyl-carbazole stock solution to 10 ml of 0.1 M sodium acetate (pH 5.0), followed by filtering through a 0.45-μm filter. Five microliters of 30% H_2_O_2 _was added to the substrate solution immediately before use.

One hundred microliters of the final substrate solution was added to each well, and the plate was incubated in the dark for 5–60 min at room temperature. The reaction was stopped by washing the wells with deionized water. The plate was air-dried overnight at room temperature in the dark and sent to ZellNet Consulting, Inc. , where the spots were enumerated automatically using an ImmunoSpot Series I analyzer (BD Biosciences). If overlapping spots (confluence) were present in the wells, the number of spots in a nonconfluent area of that well was determined. To estimate the total number of spots in each well with confluence, the following equation was used: total spot number = spot count + 2 × (spot count × % confluence / [100% - % confluence]).

### Statistical analysis

Student's *t *test was performed using Prism 4.0 Graphpad Software (San Diego, CA, USA). Statistical analysis, power analysis and the sample size per group were evaluated and found to be statistically acceptable by Dr Lyle Broemling (Associated Professor of Biostatistics, The University of Texas MD Anderson Cancer Center).

## Results

### Induction of antigen-specific IgG by vaccination with VRP-*neu*

**Figure 1:**
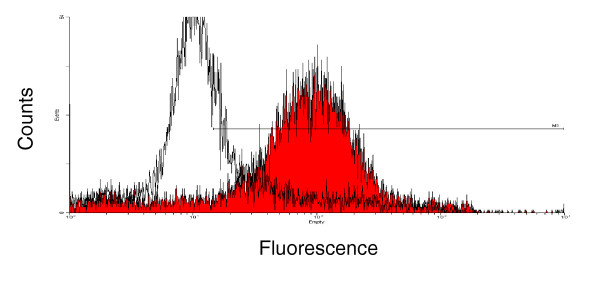
Flow cytometric analysis of A2L2 cells with serum from mice vaccinated with virus-like replicon particles (VRP)-*neu *or VRP-hemagglutinin (HA). Serum was collected 2 weeks after a single vaccination of Balb/c mice with 10^6 ^IU VRP-*neu *(filled curve) or 10^6 ^IU VRP-HA (open curve). The primary serum was diluted with PBS (1:100), and FITC-labeled goat anti-mouse IgG diluted in PBS (1:1000) was used as the secondary antibody.

Groups of Balb/c mice (*n *= 5 per group) were vaccinated once subcutaneously in one hind leg footpad with either 10^6 ^IU VRP-*neu *or 10^6 ^IU VRP-HA suspended in PBS. The HER2/*neu*-specific humoral response of serum pooled from mice in each group was measured 14 days later by flow cytometry using A2L2 cells. Compared with the mice vaccinated with VRP-HA, the mice vaccinated with VRP-*neu *had a strong IgG response (Fig. 1). Pre-immune sera for both groups were nonreactive with A2L2 cells, and immune sera from both vaccinated groups were nonreactive with 66.3 cells, the parental cell line from which A2L2 was derived by transfection with *neu *(data not shown).

### Protection from tumor challenge in a mammary fat pad prevention model following vaccination with VRP-*neu*

**Figure 2:**
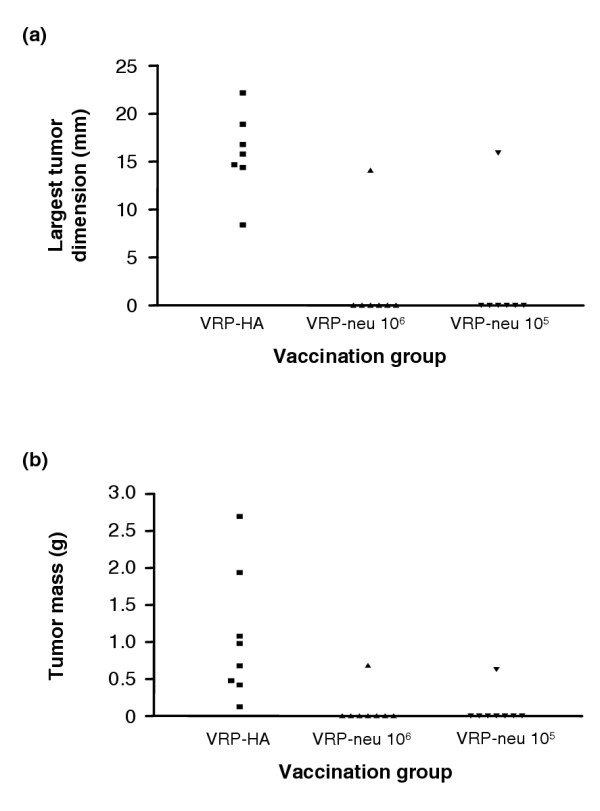
Protection from tumor challenge after vaccination with virus-like replicon particles (VRP)-*neu*. Groups of seven or eight mice were vaccinated three times at 2-week intervals with VRP-*neu *or VRP-hemagglutinin (HA). Two weeks after the final vaccination, the mice were challenged with A2L2 cells injected into a mammary fat pad. Five weeks after the tumor challenge, **(a) **the largest dimension of each tumor was measured and **(b) **the mice were killed and the mass of the tumor in each animal was determined.

Groups of mice (*n *= 7 per group) were vaccinated subcutaneously with 10^5 ^IU or 10^6 ^IU VRP-*neu *or with 10^6 ^IU VRP-HA three times at 14-day intervals. Two weeks after the final vaccination, the mice were challenged with 2.5 × 10^4 ^A2L2 cells injected into a mammary fat pad. Five weeks after tumor challenge, the largest tumor dimension was measured and the mice were killed. If a tumor was present, its mass was determined. All of the mice vaccinated with VRP-HA had a measurable tumor, whereas only one mouse in each group vaccinated with 10^6 ^IU VRP-*neu *or 10^5 ^IU VRP-*neu *had a measurable tumor (Fig. 2a,2b). These findings clearly demonstrate that vaccination three times with either 10^5 ^IU or 10^6 ^IU VRP-*neu *protected mice from challenge with A2L2 cells. VRP-HA failed to provide protection for any of the mice, and therefore the protective effect was specific for the vaccine containing the gene for HER2/*neu*.

### Determination of the minimal effective vaccine dose in two tumor prevention models

**Figure 3:**
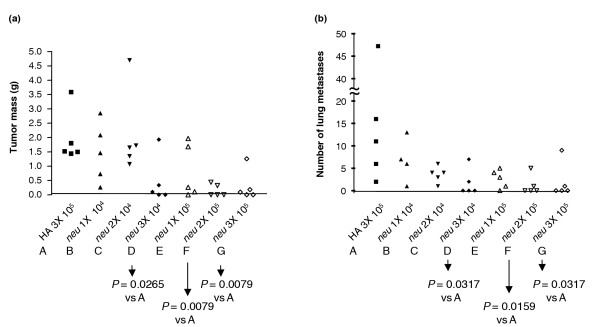
Minimal effective vaccine dose in two tumor prevention models. Groups of four or five mice were vaccinated once, twice or three times (1X, 2X, or 3X) at 2-week intervals with virus-like replicon particles (VRP)-*neu *or VRP-hemagglutinin (HA) (10^4 ^IU or 10^5 ^IU). Two weeks after the final vaccination, the mice were challenged with A2L2 cells injected into a mammary fat pad or intravensouly. The mice were killed 5 weeks after the tumor challenge. **(a) **The mass of the mammary tumors, if present, was determined in the mammary fat pad prevention model group, and **(b) **the number of surface metastases on the lungs was determined in the experimental lung metastasis prevention model group.

Because vaccination three times with 10^5 ^IU VRP-*neu *prevented tumor growth in a mammary fat pad, we next determined the minimum number of VRP-*neu *particles and the minimum number of vaccinations necessary to significantly inhibit tumor growth. In the mammary fat pad prevention model, vaccination twice with 10^5 ^IU VRP-*neu *or vaccination three times with 10^4 ^IU VRP-*neu *completely prevented tumor growth in many mice and significantly reduced the tumor mass in the entire group compared with the tumor mass of the mice vaccinated three times with VRP-HA (Fig. 3a). Identical results were obtained in the experimental lung metastasis prevention model, in which mice were injected with A2L2 cells intravenously in the tail vein after vaccination (Fig. 3b). These results demonstrate that, in both tumor models, vaccination three times with 10^4 ^IU VRP-*neu *or twice with 10^5 ^IU VRP-*neu *significantly reduced the tumor mass and lung metastasis. In addition, several mice in each vaccinated group were tumor free in mammary tissue or lungs.

### Vaccination of MMTV-c-*neu *transgenic mice

**Figure 4:**
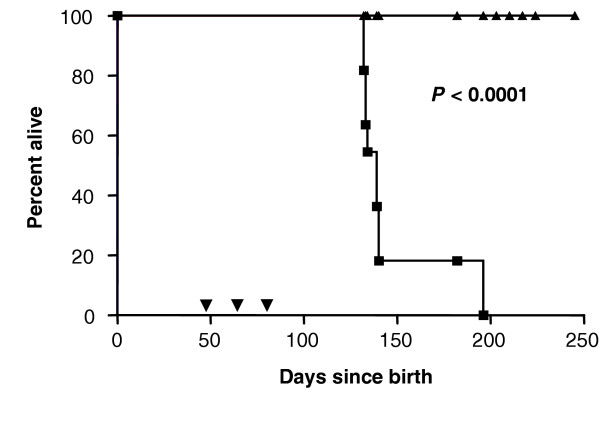
Inhibition of spontaneous tumor formation by vaccination with virus-like replicon particles (VRP)-*neu*. Groups of MMTV-c-*neu *transgenic mice (*n *= 10 per group) were vaccinated three times (▼) at 2-week intervals with 10^6 ^IU VRP-*neu *(▲) or 10^6 ^IU VRP-hemagglutinin (HA) (■). The mice vaccinated with VRP-HA were killed when moribund, and the mice vaccinated with VRP-*neu *were killed at 240 days.

MMTV-c-*neu *transgenic mice contain the activated rat *neu *gene under the control of the MMTV promoter and spontaneously develop *neu*^+ ^breast tumors within 110–120 days [45]. Without intervention, all of the mice die of breast cancer. We vaccinated groups of mice (*n *= 10 per group) three times at 14-day intervals with 10^6 ^IU VRP-*neu *or 10^6 ^IU VRP-HA and determined the effect on survival. Eight of the 10 mice vaccinated with VRP-HA were killed by 140 days owing to moribundity, and the remaining two mice were killed on day 195 (Fig. 4). None of the mice vaccinated with VRP-*neu *showed any sign of illness at 240 days, and breast tumors were not evident on palpation. The mice in the VRP-*neu*-vaccinated group were killed at this time, and gross pathologic examination of the breasts following retraction of the skin did not reveal any tumors. These results demonstrate that vaccination with VRP-*neu *prevented spontaneous formation of tumor in the breasts of *neu *transgenic mice and that tolerance to the *neu *transgene was broken by vaccination with VRP-*neu*.

### Induction of antigen-specific IgG by vaccination of MMTV-c-*neu *transgenic mice with VRP-*neu*

**Figure 5:**
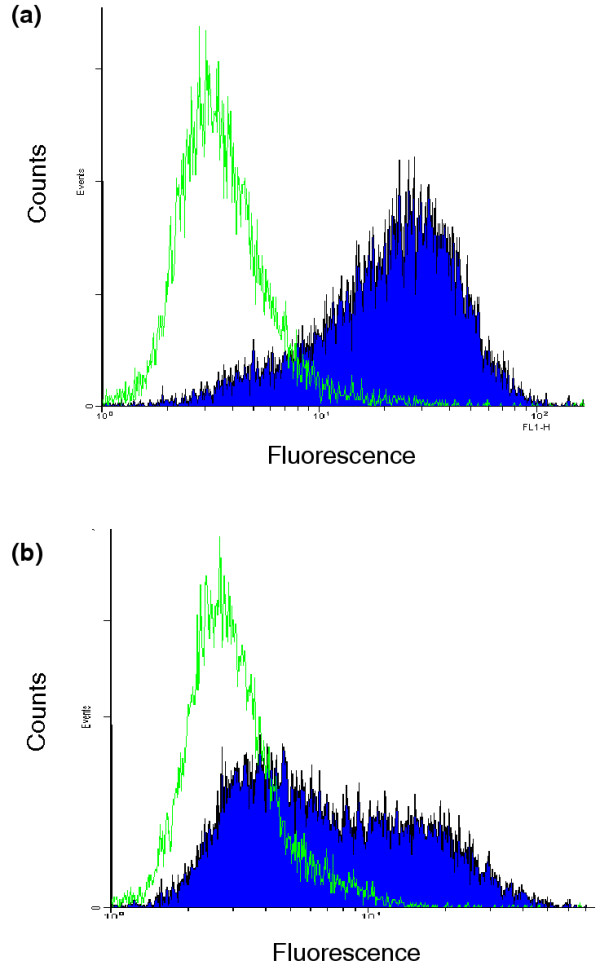
Flow cytometric analysis of A2L2 cells with sera from MMTV-c-*neu *mice vaccinated with virus-like replicon particles (VRP)-*neu *or VRP-hemagglutinin (HA). Immune serum from the mice (described in Fig. 4) was drawn at 133 days. The serum was analyzed at dilutions of **(a) **1:25 and **(b) **1:100. Filled curve, sera from VRP-*neu*-vaccinated mice; open curve, sera from VRP-HA-vaccinated mice. The secondary antibody, FITC-labeled goat anti-mouse IgG, was diluted in PBS (1:1000).

Immune serum was drawn from mice described at 133 days or 55 days after the third vaccination with VRP-*neu *or VRP-HA (Fig. 4). Flow cytometric analysis of the immune sera using A2L2 cells demonstrated that vaccination with VRP-*neu *induced a strong IgG response that was evident at dilutions of 1:25 (Fig. 5a) and 1:100 (Fig. 5b). Vaccination with the control VRP-HA failed to induce a *neu*-specific antibody response (Fig. 5a,5b). These findings indicate that vaccination with VRP-*neu *induced humoral immunity to the protein product of the *neu*-transgene in *neu*-transgenic mice, thus breaking any existing tolerance to p185.

### Tetramer analysis of CD8^+ ^T cells following vaccination with VRP-*neu *or VRP-HA

Ikuta and colleagues [43] identified a K(d)-restricted peptide for mouse HER2/*neu*. The identical sequence (PYVSRLLGI) is present in rat HER2/*neu*. We therefore ordered a tetramer containing this sequence from the National Institutes of Health Tetramer Facility at Emory University. A K(d) tetramer specific for A/PR/8/34 influenza HA was used as a positive control for VRP-HA-vaccinated mice. Balb/c mice were vaccinated three times at 2-week intervals with VRP-*neu *or VRP-HA, and the spleens from two mice were collected 3 weeks after the third injection. We found that 2.38% of the pooled spleen cells from the mice that had been vaccinated three times with VRP-*neu *were stained by both the anti-CD8 antibody and the HER2/*neu *tetramer (Table 1). This value is in excellent agreement with the percentage of dual-positive cells from mice that had been vaccinated with VRP-HA and stained with the HA tetramer (2.79%). In contrast, the percentage of dual-positive cells from the mice that had been vaccinated with VRP-HA and stained with the HER2/*neu *tetramer was only 0.22%, and the percentage from the mice that had been vaccinated with VRP-*neu *and stained with the HA tetramer was only 0.37%. These results clearly demonstrate that vaccination with VRP-*neu *produced antigen-specific CD8^+ ^T cells.

### Intracellular IFN-γ analysis of CD8^+ ^T cells following vaccination with VRP-*neu *or VRP-HA

**Figure 6:**
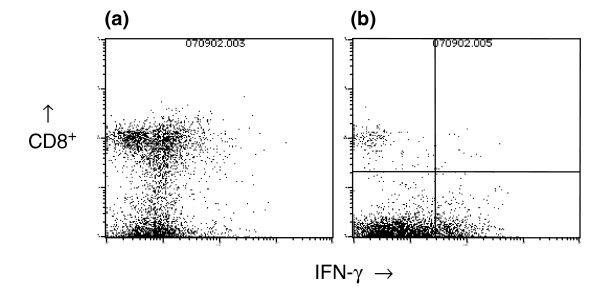
Intracellular interferon (IFN)-γ analysis of spleen cells from vaccinated mice. Spleen cells from mice vaccinated three times with **(a) **virus-like replicon particles (VRP)-*neu *or **(b) **VRP-hemagglutinin (HA) were analyzed for the presence of intracellular IFN-γ after *in vitro *culture with a *neu*-specific peptide.

An alternative assay to tetramer analysis is measurement of the percentage of CD8^+ ^T cells that also contain intracellular IFN-γ after *in vitro *stimulation with an antigenic peptide. We therefore vaccinated Balb/c mice three times at 2-week intervals with VRP-*neu *or VRP-HA, and then removed the spleens 3 weeks after the third injection. The *in vitro *culture procedure was similar to that used for tetramer analysis except that the peptide PYVSRLLGI was cultured directly with the immune spleen cells rather than with naïve spleen cells. We found that 2.80% of the spleen cells from the mice that had been vaccinated three times with VRP-*neu *stained positive for intracellular IFN-γ (Fig. 6a and Table 2) compared with only 0.27% for spleen cells from mice vaccinated with VRP-HA (Fig. 6b and Table 2). This percentage (2.80%) is in excellent agreement with that of tetramer-positive cells after vaccination with VRP-*neu *(2.38%) (Table 1).

### Analysis of immune spleen cells by IFN-γ ELISPOT after vaccination with VRP-*neu *or VRP-HA

We vaccinated Balb/c mice three times at 2-week intervals with VRP-*neu *or VRP-HA and removed the spleens 3 weeks after the third injection. The number of spleen cells secreting IFN-γ in response to overnight co-culture with A2L2 cells in an ELISPOT assay were then determined. At all three effector-to-stimulator ratios, vaccination with VRP-*neu *resulted in a statistically significant increase in the number of spots per 10^6 ^spleen cells (Table 3). This finding demonstrates that secretion of IFN-γ in response to co-culture with A2L2 cells was dependent on vaccination with VRP-*neu *and did not result from vaccination with the control VRP-HA.

## Discussion

Although more than a decade has elapsed since the original descriptions of gene vaccines [46,47], a clinically approved gene vaccine for either an infectious disease or for cancer has yet to be developed. Gene vaccines containing elements of the Alphaviruses VEE, Sindbis virus and Semliki Forest virus offer substantial clinical potential and safety [22,27]. Vaccine vectors incorporating genetic elements of Alphaviruses can be divided into two major categories [48]: expression plasmids containing viral genes that are essential for replication and transcription of the positive-strand RNA genome of the viruses [18,24], and infectious but replication-incompetent viral particles in which the genes for the viral structural proteins have been replaced by the gene for a protein antigen [20-22]. We have previously described a gene vaccine for HER2/*neu *of the first category [17]. In the present article, we describe results of a gene vaccine for HER2/*neu *of the second category.

We report here that VRP vaccine vectors derived from an attenuated strain of VEE containing the gene for rat HER2/*neu *were highly immunogenic when used to vaccinate both conventional mice and mice transgenic for the rat *neu *gene. Immune serum from mice vaccinated once with VRP-*neu *was reactive with A2L2 cells, demonstrating induction of an antigen-specific IgG response, whereas serum from mice that had been vaccinated with VRP-HA was nonreactive (Fig. 1).

To determine whether vaccination with VRP-*neu *could protect mice from challenge with a breast cancer cell line engineered to overexpress HER2*/neu*, mice that had been vaccinated three times with either VRP-*neu *or VRP-HA were injected in a mammary fat pad with A2L2 cells. Vaccination with either 10^5 ^IU or 10^6 ^IU VRP-*neu *protected all but one mouse in each group from developing tumors (Fig. 2). Because our previous experiment demonstrated that a single vaccination with 10^6 ^IU VRP-*neu *induced an IgG response, we next vaccinated mice once, twice or three times with 10^4 ^IU or 10^5 ^IU VRP-*neu *or the control VRP-HA. Vaccination three times with 10^4 ^IU VRP-*neu *(*P *= 0.0265) or twice with 10^5 ^IU VRP-*neu *(*P *= 0.0079) was sufficient to prevent tumor growth of A2L2 cells injected into a mammary fat pad (Fig. 3a). Mice similarly vaccinated were also challenged with A2L2 cells injected intravenously in the tail vein. Vaccination three times with 10^4 ^IU VRP-*neu *(*P *= 0.0317) or twice with 10^5 ^IU VRP-*neu *(*P *= 0.0159) prevented tumor growth in the lungs (Fig. 3b). Some mice in the vaccine groups had no visible lung metastases 5 weeks after the tumor challenge. This finding clearly demonstrates that vaccination with VRP-*neu *prevented tumor growth in two tumor prevention models.

There is an important point to be considered regarding our experimental models. We are vaccinating mice with the gene for rat *neu *and we are challenging mice with a cell line also overexpressing the gene for rat *neu*. We must therefore consider the possibility that mice recognized rat p185 (the protein product of the HER2/*neu *gene) as a xenoantigen and that vaccination with VRP-*neu *merely boosted, but did not initiate, an immune response. We addressed this question by vaccinating rat *neu *transgenic mice, which are immunologically tolerant to rat p185. MMTV-c-*neu *transgenic mice express the rat *neu *transgene under the control of the murine mammary tumor virus promoter and spontaneously develop *neu*^+ ^breast cancer. Vaccination of these transgenic mice with VRP-*neu *very clearly demonstrated (*P *< 0.0001) that the mice survived to 240 days old (Fig. 4), at which time the experiment was concluded. On postmortem examination, no tumor was detected in the breast of any mouse that had been vaccinated with VRP-*neu*. All mice in the control group that had been vaccinated with VRP-HA were moribund owing to extensive breast cancer by 200 days. This result is much more dramatic than our previous finding that vaccination of MMTV-c-*neu *transgenic mice with the Sindbis/DNA plasmid-based ELVIS replicon vector increased survival of the transgenic mice but failed to protect against tumor formation and death [17]. To determine whether vaccination of the MMTV-c-*neu *transgenic mice with VRP-*neu *induced an IgG response, as we found in Balb/c mice (Fig. 1), we tested the serum of the vaccinated *neu*-transgenic mice at 133 days old. Vaccination with VRP-*neu *induced a strong, antigen-specific IgG response in these transgenic mice (Fig. 5). Vaccination with VRP-*neu *therefore overcame tolerance to p185 (Fig. 5).

In our present study, we performed three separate *in vitro *T-cell assays to determine whether vaccination with VRP-*neu *induces antigen-specific T cells. In the first assay we used tetramers containing an antigenic peptide of HER2/*neu *to analyze spleen cells from mice vaccinated with VRP-*neu*. As a positive control we used a tetramer containing an antigenic peptide of HA. We found that 2.38% of the CD8^+ ^spleen cells resulting from vaccination with VRP-*neu *were positive for tetramer binding (Table 1), a value in excellent agreement with the 2.79% of the spleen cells from VRP-HA-vaccinated mice that were positive for an HA-specific tetramer. We further analyzed the induction of antigen-specific CD8^+ ^T cells resulting from VRP-*neu *vaccination by performing intracellular IFN-γ analysis. We found that 2.80% of CD8^+ ^cells from VRP-*neu*-vaccinated mice were positive for intracellular IFN-γ (Table 2), a value in excellent agreement with the 2.38% tetramer-binding cells (Table 1). In the third assay we measured the number of IFN-γ secreting cells by ELISPOT analysis (Table 3). At all three effector:stimulator ratios, the number of IFN-γ secreting cells in the VRP-*neu*-vaccinated spleen cells was significantly greater than that in the VRP-HA-vaccinated spleen cells.

These three independent assays clearly demonstrate that vaccination with VRP-*neu *induced antigen-specific CD8^+ ^T lymphocytes. The tetramer and intracellular IFN-γ assays further indicate that immune spleen cells were able to recognize an antigenic peptide of p185 presented on the A2L2 cells used for the ELISPOT assay.

The *in vivo *and *in vitro *experiments described demonstrate that vaccination induced antigen-specific cell-mediated and humoral immunity, but they do not indicate the role that each type of immunity may have played in the resulting antitumor effect. Therefore, although vaccination with VRP-*neu *produced antigen-specific IgG in both conventional and transgenic mice, whether this antibody played a role in the antitumor effect remains unclear. Pilon and colleagues [49] reported that antibody was not required for the antitumor effect of a plasmid vaccine against HER2/*neu*. Lindencrona and colleagues [50] similarly induced antitumor immunity against HER2/*neu *in B cell-deficient mice. Although T-cell immunity alone may be sufficient in tumor prevention models in mice, a clinical trial showed that trastuzumab clearly benefited patients and increased the antitumor effect of a whole-cell vaccine to HER2/*neu *[51].

## Conclusion

Our findings demonstrate that vaccination with VRP-*neu *inhibited or eliminated tumor growth in prevention models in which breast tumor cells had been injected either in the mammary fat pad or intravenously. Vaccination with VRP-*neu *also prevented tumorigenesis in transgenic mice in which the *neu *gene was expressed in the breasts under the control of an MMTV promoter. Vaccination with VRP-*neu *induced antigen-specific CD8^+^, and this finding corresponded with the absence or inhibition of tumor growth. Furthermore, vaccination with VRP-*neu *induced antigen-specific IgG in both conventional and transgenic mice, and tolerance to HER2/*neu *in *neu*-transgenic mice was broken by vaccination with VRP-*neu*. These findings suggest that VRP-*neu *constitutes a powerful gene vaccine that induces both cellular and humoral immunity against HER2/*neu*. We speculate that such vaccination could be more effective than passive immunotherapy using monoclonal antibodies such as trastuzumab in patients with breast cancer.

## Abbreviations

BSA = bovine serum albumin; FACS = fluorescence-activated cell sorting; FCS = fetal calf serum; FITC = fluorescein isothiocyanate; HA = hemagglutinin; IFN = interferon; IU = infectious units; MEM = modified Eagle's medium; MMTV = mouse mammary tumor virus; PBS = phosphate-buffered saline; VEE = Venezuelan equine encephalitis virus; VRP = virus-like replicon particles.

## Competing interests

MFM is an employee of AlphaVax, Inc. and has options to purchase the company's stock. None of the other authors have any financial interest in AlphaVax, Inc. or in any products that could result from this research. All of the research was conducted independently at the University of Texas MD Anderson Cancer Center and was not influenced in any way by employees or the management of AlphaVax, Inc.

## Authors' contributions

The authors' contributions to this research are reflected in the order shown with the exception of LBL, who supervised all aspects of this research and the preparation of this report.

## Figures and Tables

**Table 1:** Tetramer analysis of CD8^+ ^T cells after vaccination three times with virus-like replicon particles (VRP)-*neu *or VRP-hemagglutinin (HA)

Vaccination	CD8^+ ^cells (%)	Tetramer-positive CD8^+ ^cells (%)	Tetramer-positive CD8^+^/CD8^+ ^(%)
*neu*-specific tetramer			
VRP-*neu*	15.98	0.38	2.38
VRP-HA	23.06	0.05	0.22
HA-specific tetramer			
VRP-*neu*	16.01	0.06	0.37
VRP-HA	17.46	0.50	2.79

**Table 2:** Intracellular interferon-γ analysis of CD8^+ ^T cells after vaccination three times with virus-like replicon particles (VRP)-*neu *or VRP-hemagglutinin (HA)

Vaccination	Isotype control (%)	Interferon-γ^+^/CD8^+ ^(%)
VRP-*neu*	0.10	2.80
VRP-HA	0.14	0.27
Naïve	0.03	0.39

**Table 3:** Interferon-γ ELISPOT assay after vaccination with virus-like replicon particles (VRP)-*neu *or VRP-hemagglutinin (HA)

Effector:stimulator	VRP-*neu * (spots/10^6 ^cells)	VRP-HA (spots/10^6 ^cells)
100:1	99^a^	11
50:1	126^a^	17
25:1	129^a^	25
Effector only	2	5
Concanavalin A	256^a,b^	Not determined

^a^*P *= 0.05 by Student's *t *test.

^b^Spleen cells stimulated with concanavalin A as a positive control for the assay.
